# A Novel Ornithine Aminotransferase Splice Site Mutation Causes Vitamin B6-Responsive Gyrate Atrophy

**DOI:** 10.18502/jovr.v19i1.15446

**Published:** 2024-03-14

**Authors:** Samira Molaei Ramshe, Safoura Zardadi, Elham Alehabib, Ramin Nourinia, Javad Jamshidi, Mohsen Soosanabadi, Hossein Darvish

**Affiliations:** ^1^Heersink School of Medicine, University of Alabama at Birmingham, Birmingham, AL, USA; ^2^Department of Biology, School of Basic Sciences, Science and Research Branch, Islamic Azad University, Tehran, Iran; ^3^Student Research Committee, Department of Medical Genetics, School of Medicine, Shahid Beheshti University of Medical Sciences, Tehran, Iran; ^4^Ophthalmic Research Center, Research Institute for Ophthalmology and Vision Science, Shahid Beheshti University of Medical Sciences, Tehran, Iran; ^5^Noncommunicable Diseases Research Center, Fasa University of Medical Sciences, Fasa, Iran; ^6^Neuroscience Research Australia, Randwick, Sydney, NSW, Australia; ^7^Department of Medical Genetics, Semnan University of Medical Sciences, Semnan, Iran; ^8^Neuroscience Research Center, Faculty of Medicine, Golestan University of Medical Sciences, Gorgan, Iran

**Keywords:** Gyrate Atrophy, Mutation, Ornithine Aminotransferase, Vitamin B6

## Abstract

**Purpose:**

Gyrate atrophy of the choroid and retina (GACR) is a rare congenital disorder and mutations in the ornithine aminotransferase (OAT) gene has been specified as the underlying cause. Patients show a high level of ornithine in body fluids which may be controlled by low protein diets. Pyridoxine (vitamin B6) supplementation may also be effective, however, most patients appear to be nonresponsive to this modality of treatment.

**Case Report:**

Here, we report a characterized case of a vitamin B6-responsive GACR who had a splicing mutation in the *OAT* gene. The GACR diagnosis was confirmed through the clinical features, imaging, biochemical findings, and whole-exome sequencing (WES) results. WES data revealed the splicing mutation in intron 4 of the *OAT* gene (NM_001322967: c.425-1G > A).

**Conclusion:**

Our knowledge about the diagnosis and treatment of GACR can be improved by identifying novel mutations in the *OAT* gene and accurate follow-up of the patients to determine how they respond to treatment.

##  INTRODUCTION

Gyrate atrophy of the choroid and retina (GACR-OMIM: 258870) is a rare autosomal recessive ophthalmic disorder with the highest incidence in Finland.^[1]^ GACR is a phenotypically variable disorder with inconsistent age of onset (childhood–40s). Night blindness and constriction of the visual field which are caused by multiple round areas of chorioretinal atrophy in the periphery are the first complaints noticed in patients. Progression of the atrophic areas leads to central visual loss after 40 years of age.^[2]^ Ocular symptoms are the major manifestation in most GACR patients; however, type II fibers atrophy, muscle weakness, intellectual disability, neonatal hyperammonemia, and peripheral nervous system abnormalities have also been reported in some cases.^[3,4]^

GACR is caused by homozygous or compound heterozygous mutations in the *OAT* gene.^[5]^ The *OAT* gene, which consists of 11 exons, encodes the mitochondrial enzyme ornithine aminotransferase which is a key enzyme in the pathway that converts arginine and ornithine into the neurotransmitters glutamate and Gamma-aminobutyric acid (GABA); where vitamin B6 works as a co-factor for the enzyme.^[6]^

OAT enzyme deficiency caused by *OAT *gene mutations leads to significant elevation of ornithine concentration in plasma (about 10 fold) and other body fluids. Long-term exposure to high concentrations of ornithine in the blood causes retinal toxicity in patients along with gyrate atrophy of the choroid and retina.^[2,6]^ Introduction of a low-protein diet and vitamin B6 therapy can slow the progression of chorioretinal degeneration and both have been recommended as treatment for all newly diagnosed patients with gyrate atrophy.^[7,8,9,10]^

Molecular analysis for the detection of *OAT* mutations is recommended for accurate diagnosis of disease and determination of the vitamin B6-responsive variations.

Here, we present an Iranian male patient diagnosed with GACR. Whole-exome sequencing (WES) detected a splicing mutation, c.425-1G > A in the *OAT* gene. In addition, we have summarized the entire list of reported *OAT* mutations that correlate with GACR patients as a literature review.

##  CASE REPORT 

**Figure 1:**
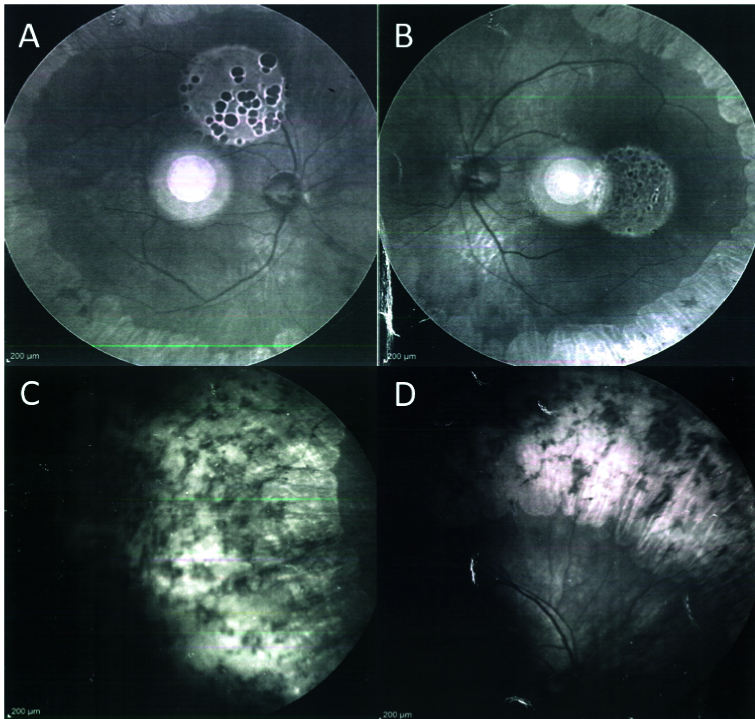
Infrared reflectance imaging (IR) shows sharply defined, scalloped retina pigment epithelium and choroidal atrophy areas in the mid-peripheral zone (A, B, C, D).

The patient was a 24-year-old Mazandarani male from a healthy and non-consanguineous family. He signed the consent form and allowed the publishing of the results of his examination and tests. The Ethical Committee of the Shahid Beheshti University of Medical Sciences approved all study procedures. The patient presented with gradual visual loss, which was first noted when he was five years old. He also had a history of decreased night vision since the age of 15. His best-corrected visual acuity in each eye was 4/10. The refractive error was –13.5–0.75 × 50 for his right eye and –13.5–1.00 × 150 for his left eye. Anterior segment examination showed mild posterior subcapsular cataracts in both eyes. Fundus examination of both eyes revealed sharply demarcated areas of the choroidal and retinal atrophy in gyrate shape involving the mid periphery, however, the macula was not affected [Figure 1]. An electroretinogram demonstrated a reduction of both cone and rod responses.

Furthermore, biochemical tests showed elevation of plasma ornithine levels (1463.2 micmol/L; Normal range: 27–83 micmol/L). Introduction of a low protein diet and vitamin B6 therapy significantly decreased the amount of ornithine to 818.8 micmol/L after three months. DNA sequencing revealed a mutation in the *OAT* gene and confirmed the ophthalmological examination, imaging, and biochemical test findings for gyrate atrophy diagnosis.

### DNA Sequencing and Analyses

DNA was extracted from peripheral blood of the patient and his parents using the salting-out method. After quality and concentration assessment of the DNA samples, they were subjected to WES. WES enrichment was performed using an Agilent Sure Select V6 Target Enrichment Kit, and the library was sequenced on the Illumina Hiseq 4000 platform, performed by Macrogen, South Korea. All exons and flanking 10 bps were detected and analyzed. Then, rare variants (MAF < 0.01) were analyzed in silico for pathogenicity predicting using predictor tools databases. The final assessment from WES data revealed a homozygote splicing mutation in the last nucleotide of the intron 4 of the *OAT* gene (NM_001322967: c.425-1G > A).

The mutation novelty information was ascertained by filtering throughout the following databases: ExAC (http://exac.broadinstitute.org/); genomAD (https://gnomad.broadinstitute.org/); dbSNP137 (https://www.ncbi.nlm.nih.gov/snp/); and 1000Genome projects (http://www.internationalgenome.org). In addition, the Iranome database was accessed as a local reference for exome variants (http://www.iranome.ir/). The mutation was predicted to be pathogenic and damaging according to different prediction tools such as DANN score, mutation taster, FATHMM, SIFT, PROVEN, and REVEL database.

**Figure 2:**
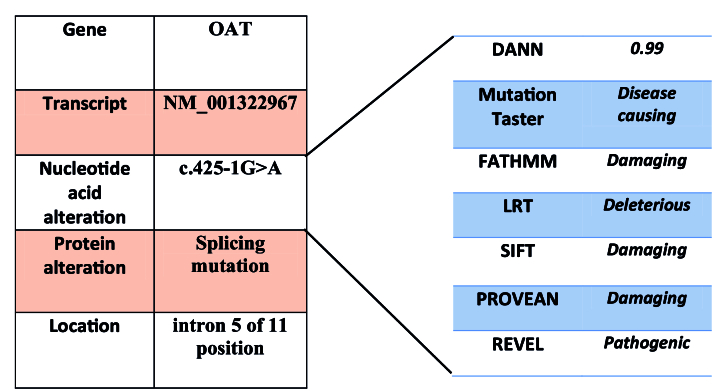
Family pedigree and partial sequence of OAT gene (genotypes of mother and father have been detected as heterozygotes in c.425-1; however, the proband's nucleotide sequencing has shown the homozygous nucleotide variation in this position). The site of mutation is shown by the arrow.

To confirm the WES result, primer was designed to amplify a short sequence containing the targeted mutation using PCR. Then Sanger sequencing was performed in both forward and reverse directions for the amplified sequence. Also, the patient's parents were investigated and both were heterozygous for the mentioned mutation [Figure 2].

### Search Method of Literature Review

In this study, a review in genotype and phenotype of previously reported GACR cases was conducted up to June 2020, which is summarized in Table 1. We used PubMed (https://pubmed.ncbi.nlm.nih.gov) and Google Scholar (http://scholar.google.com) databases to search published papers and abstracts using the following keywords: OAT mutation, Gyrate Atrophy, and GACR, and also Google using keywords: OAT OMIM, Clinvar OAT, and LOVD OAT. Only studies published in English were included. The following information was extracted from each article: Nucleotide change, Amino acid change, Mutated exon/intron, Mutation type, Zygosity status, Age of onset (years), Clinical symptoms, Plasma ornithine levels (µmol/L), Response to vitamin B6 therapy and origin of reported cases. This table included missense, frameshift, nonsense, and splicing mutations, however, large structural indels were excluded.

##  DISCUSSION

**Figure 3:**
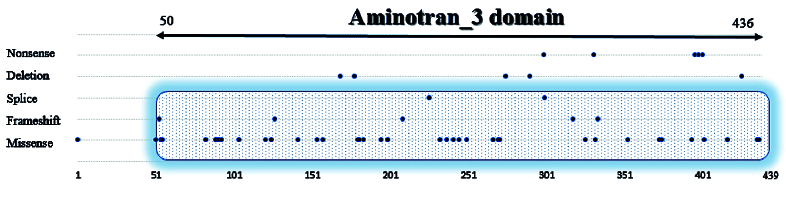
Distribution of the *OAT* gene mutations on OAT protein. This figure shows OAT protein domains and distribution and numbers of mutations in OAT domains according to their type. Aminotran_3 domain is shown as the main domain of OAT enzyme. Mutation diagram circles are colored with respect to the corresponding mutation types. In the case of different mutation types at a single position, the circle's color is determined with respect to the most frequent mutation type. Mutation types and corresponding color codes are as follows: Missense mutations (Green), truncating mutations: Nonsense, nonstop, frameshift deletion, frameshift insertion, splice site (Black), inframe mutations: Inframe deletion, inframe insertion (Brown), and splice mutations (Orange). The location of the detected mutation in our study is a site for five previously reported mutations, as shown in this figure.

GACR is a rare metabolic disorder due to ornithine aminotransferase enzyme deficiency, which in turn results in progressive vision loss, myopia, cataracts, and night blindness in patients.^[2]^ As a result of the common symptoms that exist between GACR and retinitis pigmentosa (RP), misdiagnosis is probable. Therefore, molecular and biochemical analysis could help in the differential diagnosis.^[11]^ OAT is a homohexameric enzyme which has a vital role in proline and GABA synthesis from ornithine and arginine.^[12]^ The OAT enzyme deficiency caused by different mutations in the *OAT *gene leads to hyperornithinemia and chorioretinal degeneration. To date, about 80 mutations have been reported in the *OAT* gene. Distribution of the mutations in the OAT protein is shown in Figure 3. We reviewed the literature to investigate and compare the reported mutations, the resulting phenotypes, and their response to vitamin B6 therapy [Table 1].

**Table 1:** .


**Nucleotidechange**	**Amino acidchange**	**Mutated exon/intron**	**Mutation type**	**Zygosity status**	**Age of onset (years)**	**Clinical symptoms**	**Plasma ornithine levels(µmol/L)**	**Response to vitamin B6 therapy**	**Origin**	**Reference**
c.3G > A	p.Met1Ile	3	Missense	Homozygous	2	the small, discrete areas of depigmentation appeared to be deep to the retina and were seen at high magnification and the choroidal vascular pattern was prominent/ Myopia/ Night blindness/ Cataracts	745	*NI	Lebanese	[25, 26]
c.3G > A	p.Met1Ile	3	Missense	Homozygous	9	Significantly greater severity in chorioretinal involvement/some discrete areas of chorioretinal atrophy the lesions appeared to have coalesced and formed confluent areas of atrophy encroaching upon the posterior pole of the retina/ increasing in the pigment surrounding atrophic lesions/ many of the choroidal vessels visible in the atrophic regions appeared to be devoid of blood/ The retinal vasculature appeared narrowed/ Myopia/ Night blindness/ Cataracts	876	NI	Lebanese	[25, 26]
c.3G > A	p.Met1Ile	3	Missense	Homozygous	32	Myopia/ Night blindness/ Cataracts	771	NI	Lebanese	[25, 26]
c.3G > A	p.Met1Ile	3	Missense	Homozygous	33	Myopia/ Night blindness/ Cataracts	800	NI	Lebanese	[25, 26]
c.152G > A	p.Gly51ASP	3	Missense	Homozygous	14	Abnormal retinal appearance/ increasing myopia	1152	NI	NI	^[27]^
c.152G > A/ c.1181G > A	p.Gly51Asp/ p.Cys394Tyr	3/11	Missense/ Missense	Compound heterozygous	8	Night blindness	982	Responsive	Southern Italy	^[12]^
c.152G > A/ c.1181G > A	p.Gly51Asp/ p.Cys394Tyr	3/11	Missense/ Missense	Compound heterozygous	3	Night blindness	788	Responsive	Southern Italy	^[12]^
c.159del	p.His53Glnfs*8	3	Frameshift	NI	NI	NI	NI	NI	Iraqi Jew	^[28]^
c.159del	p.His53Glnfs*8	3	Frameshift	Homozygous	28	Progressive night blindness and visual impairment/ Automated visual fields were constricted/Bilateral posterior subcapsular cataracts	697	Responsive	Syrian Jews	^[20]^
c.162C > A	p.Asn54Lys	3	Missense	Heterozygote	14	NI	769	Nonresponsive	NI	[29, 30]
c.163T > C	p.Tyr55His	3	Missense	NI	NI	NI	NI	NI	Aust/ Hung/ English	^[28]^
c.163T > C/ c.748C > T	p.Tyr55Hi/ p.Arg250*	3/7	Missense/ Nonsense	Compound heterozygous	52	Night blindness from a young age and problems with peripheral vision from early teenage years	∼ 700	NI	NI	^[27]^
c.185T > C	p.Leu62Pro	3	Missense	NI	NI	NI	NI	NI	NI	^[43]^
c.192_193del/ c.596C > A	p.Gly65Lysfs*15/ p.Pro199Gln	3/6	Frameshift/ Missense	Compound heterozygous	23	Retinal findings were characteristic and elevation of plasma ornithine	NI	Nonresponsive	Adopted	^[31]^
c.248G > A	P.Ser83Asn	3	Missense	Homozygous	19	Binocular myopia/night blindness/gradual dark blindness/ decreased central vision and visual acuity/ along with the prominent presence of fundus flecks in the retina	470.76	NI	Chinese	^[32]^
c.199+303C > G	NI	Intron 3	Non coding	NI	12	Chorioretinal degeneration/ hyperornithinemia	780	NI	Algerian	[17, 57]
c.267C > A	p.Asn89Lys	4	Missense	NI	NI	NI	NI	NI	Finnish	^[28]^
c.268C > G	p.Gln90Glu	4	Missense	NI	35	Strong myopia/ cataracts/ concentric contraction/ proximal dominant muscular atrophy/ hyperornithinemia	NI	NI	Japanese	^[24]^
c.271G > A	p.Gly91Arg	4	Missense	Homozygous	3	Hyperornithinemia/ mild mental retardation with hyperactivity/ distractibility and short attention span/ delayed language development and speech defects/ retinal periphery/ in high mydriasis, there were many round, sharply defined, whitish areas of choroid atrophy	NI	NI	Adopted	^[9]^
c.272G > A	p.Gly91Glu	4	Missense	Homozygous	10	Abnormal retinal appearance/ increasing myopia/ multiple intraretinal cystic spaces bilaterally	1390	NI	NI	^[27]^
c.278G > T	p.Cys93Phe	4	Missense	NI	NI	NI	NI	NI	German/Italian	^[28]^
c.311A > G/ c.991C > T	p.Gln104Arg/ p.Arg331*	4/9	Missense/ Nonsense	Compound heterozygous	10	NI	776	NI	Greece	^[12]^
c.362G > A/ c.897C > G	P.Gly121Asp/ P.Tyr299*	4/8	Missense/ Nonsense	Compound heterozygous	38	Abnormal retinal appearance/ increasing myopia	∼ 1000	NI	NI	^[27]^
c.373_375del/ c.978T > A	p.Glu125del/ p.Asn326Lys	4/9	In frame/ Missense	Compound heterozygous	23	Constricted visual fields	NI	Nonresponsive	Japanese	[24, 34]
c.381dup	p.Thr128Tyrfs*2	4	Frameshift	NI	NI	NI	NI	NI	Welsh	^[28]^
c.425G > A	p.Gly142Glu	5	Missense	Homozygous	24	Hyperornithinemia/ typical fundus changes of scalloped chorioretinal atrophies with sharp margins/ deteriorated dark adaptation and constricted visual fields	NI	Nonresponsive	Japanese	[24, 35]
c.425-1G > A	NI	Intron 4	Splicing	Homozygous	24	Gradual visual loss/ mild posterior subcapsular cataract / night blindness/ myopia/ Hyperornithinemia/ reduction of both cone and rod response	1463.2	Responsive	Iranian	This study & ^[15]^
c.425-2A > G	p.Val143Argfs*9	Intron 4	Splicing	NI	NI	NI	NI	NI	NI	^[38]^
c.425_520del	p.Val143Argfs*9	5	Splicing	Compound heterozygous	42	NI	1189	Nonresponsive	Danish/Swedish	^[23]^
c.425G > A/ c.199+11_ 199+16dup	p.Gly142Glu	5/Intron 2	Missense/Non coding	Compound heterozygous	19	Gradual visual loss/ progressive night vision/ bilateral posterior subcapsular cataracts/ her fundus exhibited bilateral severe chorioretinal atrophy involving the mid periphery/ leakage at the margin of chorioretinal atrophy and dye accumulation in the maculae of both eyes/ disclosed cystoid macular edema was evident in both eyes/ visual field constriction in both eyes	783	Responsive	Korean	^[19]^
c.425G > A/ c.199+11_ 199+16dup	p.Gly142Glu	5/Intron 2	Missense/Non coding	Compound heterozygous	19	Gradual visual loss/ progressive night vision/ her fundus exhibited bilateral severe chorioretinal atrophy involving the mid periphery, leakage at the margin of chorioretinal atrophy and dye accumulation in the maculae of both eyes/ disclosed cystoid macular edema was evident in both eyes/ visual field constriction in both eyes	831	Responsive	Korean	^[19]^
c.461G > A	p.Arg154His	5	Missense	Homozygous	NI	hyperammonemia in the neonatal period, raised ornithine	NI	NI	NI	^[51]^
c.461G > T	p.Arg154Leu	5	Missense	NI	NI	NI	NI	NI	English/German	^[28]^
c.473A > C	p.Tyr158Ser	5	Missense	Homozygous	67	Retinitis pigmentosa/ progressively constricting visual fields in both eyes and nyctalopia/ bilateral cataracts	586	Responsive	Honduras	^[36]^
c.472_486del	p.Tyr158_ Gly162del	5	In frame	Compound heterozygous	10	Night blindness/ myopia/ posterior subcapsular cataracts/ constriction of visual fields	NI	NI	American	^[37]^
c.498C > A	P.Tyr166*	4	Nonsense	Homozygous	16	Bilateral visual acuity reduction to 0.4 on the right eye and 0.5 on the left eye with a myopic astigmatism with aspheric, coalescent chorioretinal atrophic changes in the periphery, bilateral cystoid edema myopia	877	NI	Turkish	^[52]^
c.504_505del/ c.1276C > T	p.Lys169Aspfs*10/ P.Arg426*	5/11	Frameshift/ Nonsense	Compound heterozygous	6 (in first examination)	Night blindness/ sharply demarcated circular areas of chorioretinal atrophy in the entire peripheral retina	1041	Nonresponsive	Japanese	^[39]^
c.504_505del/ c.1276C > T	p.Lys169Aspfs*10/ P.Arg426*	5/11	Frameshift/ Nonsense	Compound heterozygous	2 (in first examination)	Retinal degeneration in the superior peripheral area in both eyes/ constricted visual fields at 7 years of age/ chorioretinal atrophy at the superior retinal arcade in the right eye at 12 years of age	952	NI	Japanese	^[39]^
c.532_536del/ c.897C > G	p.Trp178/ p.Tyr299*	5/7	Nonsense/ Nonsense	Compound heterozygous	22	Areas of chorioretinal atrophy in the periphery/ myopia/ slowly increasing blurred vision and eye floaters in both eyes/ narrow visual field	574.6	NI	NI	^[40]^
c.425-2A > G/ c.952G > A	p.Val143Argfs*9 /p.Glu318Lys	Intron 4/9	Splicing/ Missense	Compound heterozygous	29	All characteristic clinical and ophthalmological features of gyrate atrophy	NI	Responsive	NI	[8, 17]
c.425-2A > G/ c.952G > A	p.Val143Argfs*9 /p.Glu318Lys	Intron 4/9	Splicing/ Missense	Compound heterozygous	31	Constricted visual fields, abnormal electroretinography and electro-oculography/ round and gyrate areas of total vascular atrophy of peripheral choroid and retina/ elevated serum ornithine	NI	Responsive	English/ German/ Scottish	[7, 48]
c.539G > C	p.Arg180Thr	6	Missense	Homozygous	NI	NI	NI	NI	Finnish	[41, 28, 53]
c.539G > C/ c.897C > G	p.Arg180Thr/ p.Tyr299*	6/8	Missense/ Nonsense	Compound heterozygous	NI	NI	NI	Nonresponsive	Italian/ Dutch/ Irish	[31, 56]
c.542C > T	p.Thr181Met	6	Missense	Homozygous	18	A posterior subcapsular cataracts in both eyes/ more severe in the right eye/ chorioretinal atrophy with scalloped border approached to the posterior pole/ cycloscopically short and scanty ciliary processes	NI	Responsive	Japanese	[24, 42]
c.550G > A	p.Ala184Thr	6	Missense	NI	NI	NI	NI	NI	NI	^[43]^
c.550del	p.Ala184Leufs*46	6	Frameshift	NI	NI	NI	NI	NI	Portuguese	^[28]^
c.583G > T/ c.812G > A	p.Asp195Tyr/ p.Arg271Lys	6/8	Missense/ Missense	Compound heterozygous	5	Hyperornithinemia/ yellow-white spots at the peripheral fundus and an abnormal reflex in the macular area at the age of 4 years/ night blindness	NI	Nonresponsive	Japanese	[24, 42]
c.596C > A/ c.1250C > T/ c.1311G > T	p.Pro199Gln/ p.Pro417Leu/ p.Leu437Phe	6/11/11	Missense/ Missense/ Missense	Compound heterozygous	6	Night blindness	835	Partial reduction of ornithine levels	English	[12, 54]
c.596C > A/ c.1250C > T	p.Pro199Gln/ p.Pro417Leu	6/11	Missense/ Missense	Compound heterozygous	30	Abnormal retinal appearance/ increasing myopia	908	NI	NI	^[27]^
c.596C > A/ c.1250C > T	p.Pro199Gln/ p.Pro417Leu	6/11	Missense/ Missense	Compound heterozygous	34	Night vision problems/ bilateral cataract/ night blindness	∼ 900	NI	NI	[27, 28]
c.534G > A	p.Trp178*	6	Nonsense	Homozygous	NI	NI	NI	NI	NI	^[44]^
c.534G > A/ c.825G > A	P.Trp178/ p.Trp275*	6/8	Nonsense/ Nonsense	Compound heterozygous	NI	NI	NI	NI	NI	^[44]^
c.627T > A/ c.1118G > A	p.Tyr209*/ p.Gly373Glu	6/10	Nonsense/ Missense	Compound heterozygous	19	Poor night vision since early childhood/ moderate color deficiency in the left eye/ moderately advanced posterior subcapsular cataracts bilaterally/ typical total vascular atrophy of the peripheral choroid and retina	912 ± 21	Nonresponsive	English/German	^[31]^
c.521-172_ 648+772del/ c.627T > A	p.Gly175Cysfs*18/ p.Tyr209*	6/6	Frameshift/ Nonsense	Compound heterozygous	41	Mild but definite progression of choroidal atrophy visible / cataract in the left eye	NI	Nonresponsive	English/ Dutch/ German/ French	[8, 31]
c.677C > T	p.Ala226Val	7	Missense	Homozygous	NI	NI	NI	NI	Italian	^[45]^
c.677C > T/ c.901-2A > G	p.Ala226Val/ p.Ser302_ Val339del	7/9	Missense/ splicing	Compound heterozygous	NI	NI	NI	NI	NI	^[18]^
c.677C > T/ c.1192C > T	p.Ala226Val/ p.Arg398*	7/11	Missense/ Nonsense	Compound heterozygous	7	Poor vision at 4 years of age/ typical GA lesions in the periphery of the retina/ moderate myopia and mild astigmatism	652	Responsive	Australian	^[45]^
c.698A > G	p.Gln233Arg	7	Missense	Homozygous	NI	NI	NI	NI	Mexican	^[18]^
c.710G > A	p.Gly237Asp	7	Missense	Homozygous	7	Progressive loss of vision/ a large atrophied area in retina	1140NMOL/ML	Responsive	NI	^[46]^
c.722C > T	p.Pro241Leu	7	Missense	NI	NI	NI	NI	NI	German/Italian	^[28]^
c.734A > G	p.Tyr245Cys	7	Missense	NI	NI	NI	NI	NI	English	^[28]^
c.749G > C	p.Arg250Pro	7	Missense	NI	NI	NI	NI	NI	French	^[28]^
c.800C > T	p.Thr267IIe	8	Missense	NI	NI	NI	NI	NI	Ashkenazi Jew	^[28]^
c.800C > T/ c.900+1G > A	p.Thr267IIe/NI	8/Intron 7	Missense/ Splicing	Compound heterozygous	16	High myopia/ constricted visual fields/ mild posterior subcapsular cataracts/ mid periphery chorioretinal atrophic lesions/ mild cystoid macular edema	879	Responsive	Ashkenazi Jew	^[20]^
c.808G > C	p.Ala270Pro	8	Missense	NI	NI	NI	NI	NI	Portuguese	^[28]^
c.812G > A	p.Arg271Lys	8	Missense	Homozygous	24	The chorioretinal findings were characteristic of GA/ elevation of plasma ornithine	NI	Nonresponsive	Japanese	[24, 28]
c.868_870del	p.Leu290del	7	In frame	Homozygous	10	Partial vision loss and strabismus/ large retinal atrophic area/ bilateral macular oedema with numerous circular sharply limited atrophic zones in the retina/ significant attention deficit	1039	Responsive	NI	^[47]^
c.901-1G > A	NI	Intron 7	Splicing	Homozygous	3 months	Neonatal hyperammonemia/ vomiting and abnormal cycling movements at 18 d of age/reduced level of consciousness/ increased deep tendon reflexes and hypertonicity/ detection of a rise in plasma ornithine level by 3 mo of age	638.5	NI	NI	^[21]^
c.952del	p.Glu318Serfs*11	9	Frameshift	NI	NI	NI	NI	NI	Turkish	^[28]^
c.952G > A	p.Glu318Lys	9	Missense	Homozygous	15	Night blindness	728	Responsive	English	^[12]^
c.952G > A	p.Glu318Lys	9	Missense	Homozygous	17	Poor central vision	775	NI	NI	^[27]^
c.991C > T	p.Arg331*	9	Nonsense	Homozygous	5	NA	650	NI	Turkey	^[12]^
c.994G > A	p.Val332Met	9	Missense	NI	21	NI	1140	Responsive	NI	[29, 30]
c.994G > A	p.Val332Met	9	Missense	Homozygous	7	Night blindness	526	Nonresponsive	Southern Italy	[12, 56]
c.994G > A	p.Val332Met	9	Missense	Homozygous	13	Night blindness	703	Nonresponsive	Southern Italy	^[12]^
c.955C > T	p.His319Tyr	9	Missense	Compound Heterozygous	31	Progressive blindness with the characteristic ophthalmoscopic appearance of the retina and hyperornithinemia	NI	Nonresponsive	Japanese	^[49]^
c.1031del	p.Asn344Thrfs*13	10	Frameshift	NI	NI	NI	NI	NI	West African	^[28]^
c.1058G > A	p.Gly353Asp	10	Missense	NI	NI	NI	NI	NI	Spanish	^[28]^
c.1124G > C	p.Gly375Ala	10	Missense	NI	NI	NI	NI	NI	Hispanic	^[28]^
c.1171G > A	p.Trp391*	11	Nonsense	NI	NI	NI	NI	NI	NI	^[43]^
c.1180T > C	p.Cys394Arg	11	Missense	NI	NI	NI	NI	NI	English	^[28]^
c.1181G > A	p.Cys394Tyr	11	Missense	Homozygous	40	Night blindness	805	Nonresponsive	Southern Italy	^[12]^
c.1186C > T	P.Arg396*	11	Nonsense	NI	NI	NI	NI	NI	East Indian	^[28]^
c.1192C > T	p.Arg398*	11	Nonsense	Homozygous	7 weeks	Neonatal hyperammonemia/ at age 7 weeks there was a marked elevation of plasma ornithine	680	NI	Asian	^[3]^
c.1276C > T	p.Arg426*	11	Nonsense	Homozygous	16 weeks	Neonatal hyperammonemia	481	NI	Turkish	^[3]^
c.1201G > T	p.Gly401*	11	Nonsense	NI	NI	NI	NI	NI	German/ American	^[28]^
c.1205T > C	p.Leu402Pro	11	Missense	Homozygous	NI	NI	NI	NI	Finnish	[41, 28]
c.1276C > T	p.Arg426*	11	Nonsense	Homozygous	8	Decreased vision at the age of 5/myopia/ night blindness/ typical chorioretinal atrophy with scallop margins at the peripheral fundus and an abnormal reflex in the macular area/ hyperornithinemia	NI	Nonresponsive	Japanese	[24, 42]
c.1307T > A	p.Ile436Asn	11	Missense	Homozygous	6	Visual loss	627	NI	Southern Italy	^[12]^
c.1307T > A	p.Ile436Asn	11	Missense	Homozygous	8	Patches of circumferential chorioretinal atrophy in the peripheral retina of both eyes, bilateral foveal-involving CME	582	Nonresponsive	Italy	^[50]^
c.1311G > T	p.Leu437Phe	11	Missense	NI	NI	NI	NI	NI	NI	^[28]^
	
	
${\;}^{*}$ NI, no information was available in the mentioned articles										

Gradual vision loss, night blindness, and early-onset cataract were clinically diagnosed in our patient. Hyperornithinemia was the early laboratory finding in the patient and genetic study confirmed the GACR diagnosis. Hyperornithinemia was significantly decreased by treating with a low protein diet and vitamin B6 intake as a supplement.

According to the literature, only a few GACR patients have been recognized to be responsive to vitamin B6 therapy.^[13,14]^ Genotype–phenotype correlation for vitamin B6-responsive patients has not yet been determined. Here, we described a B6-responsive case of GACR in the Iranian population. Low protein diet and vitamin B6 as a medical supplement led to a 44% reduction in plasma ornithine levels after three months and decreased adverse ocular changes in the long term.

The patient had a homozygote splice mutation in the *OAT* gene in the 3 ' splice acceptor site (AG) of intron 4 (c.425-1G > A). This mutation might be a common mutation among Iranian GACR patients as it was also reported in an 18-year-old male in a study performed by Jalali et al.^[15]^ In similar studies, other splicing mutations were reported before in the* OAT* gene.^[16,17,18,19,20,21]^ Splice site mutations can disrupt the binding of splicing enhancers, silencers, and spliceosome elements and lead to exon skipping. They primarily result in an aberrant transcript and a truncated protein.^[22]^ A 9-bp deletion (c.425-4_429del) spanning 3 ' -acceptor of exon 5 of OAT has been reported in a Danish/Swedish GACR patient in a study by McClatchey et al.^[23]^ This mutation resulted in the exon 5 skipping without any disruption in the reading frame. An A to G substitution at the 3 ' splice acceptor site of intron 4 (c.425-2A > G) was reported in the* OAT* gene in a study by Mashima et al. They also identified the exon 5 skipping in the mRNA in another study.^[17]^ Therefore, the mutation in our patient (c.425-1G > A) probably caused the same result, which is exon 5 skipping, loss of 32 amino acid residues and generating a truncated OAT enzyme. The truncated enzyme is possibly an inactive one that is not functional in the ornithine metabolic pathway and therefore has led to the GACR phenotype in our patient. The mutation's location and its vicinity has been reported for other mutations in previous studies. c.425G > A mutation was found in several studies.^[18,23,34]^ Kim et al reported the c.425G > A mutation in a pair of 19-year-old Korean female identical twins whose clinical manifestations were consistent with GACR. These two patients were also responsive to B6 therapy.^[19]^

Early diagnosis of patients with GACR, specifically those who respond positively to a low protein diet and vitamin B6 supplement intake, can significantly help in the successful treatment of the condition. Therefore, identifying OAT mutations that are responsive to treatment and determination of the respective genotype–phenotype correlation in GACR patients is essential.

##  Financial Support and Sponsorship

None.

##  Conflicts of Interest

None.
